# 4-Chloro-*N*-(3,4-dimethyl­phen­yl)benzene­sulfonamide

**DOI:** 10.1107/S1600536810015394

**Published:** 2010-05-08

**Authors:** B. Thimme Gowda, Sabine Foro, P. G. Nirmala, Hartmut Fuess

**Affiliations:** a Department of Chemistry, Mangalore University, Mangalagangotri 574 199, Mangalore, India; b Institute of Materials Science, Darmstadt University of Technology, Petersenstrasse 23, D-64287 Darmstadt, Germany

## Abstract

In the title compound, C_14_H_14_ClNO_2_S, the angle between the sulfonyl and aniline benzene rings is 65.5 (1)°. The crystal structure features inversion dimers linked by pairs of N—H⋯O hydrogen bonds. The dimethyl­phenyl ring is disordered over two different orientations approximately related by a 180° rotation about the C—N bond, with occupancies of 0.643 (6) and 0.357 (6).

## Related literature

For the preparation of the title compound, see: Shetty & Gowda (2005 ▶). For our studies of the effect of substituents on the structures of *N*-(ar­yl)aryl­sulfonamides, see: Gowda *et al.* (2009 ▶, 2010 ▶). For related structures, see: Gelbrich *et al.* (2007 ▶); Perlovich *et al.* (2006 ▶).
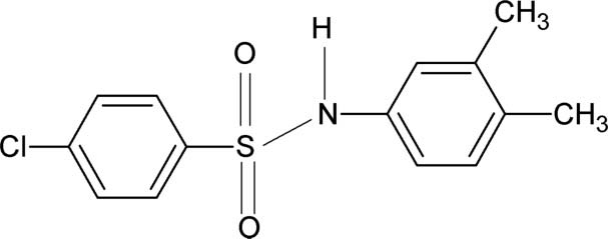


## Experimental

### 

#### Crystal data

C_14_H_14_ClNO_2_S
*M* *_r_* = 295.77
Monoclinic, 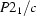

*a* = 13.5000 (9) Å
*b* = 12.4039 (8) Å
*c* = 8.7436 (7) Å
β = 104.492 (7)°
*V* = 1417.55 (17) Å^3^
*Z* = 4
Mo *K*α radiation
μ = 0.41 mm^−1^
*T* = 100 K
0.44 × 0.34 × 0.22 mm

#### Data collection

Oxford Diffraction Xcalibur diffractometer with a Sapphire CCD detector
Absorption correction: multi-scan (*CrysAlis RED*; Oxford Diffraction, 2009 ▶) *T*_min_ = 0.839, *T*_max_ = 0.915
5943 measured reflections
2890 independent reflections
2309 reflections with *I* > 2σ(*I*)
*R*_int_ = 0.015

#### Refinement

*R*[*F* ^2^ > 2σ(*F* ^2^)] = 0.039
*wR*(*F* ^2^) = 0.107
*S* = 1.07
2890 reflections
194 parameters
28 restraints
H atoms treated by a mixture of independent and constrained refinement
Δρ_max_ = 0.31 e Å^−3^
Δρ_min_ = −0.24 e Å^−3^

### 

Data collection: *CrysAlis CCD* (Oxford Diffraction, 2009 ▶); cell refinement: *CrysAlis RED* (Oxford Diffraction, 2009 ▶); data reduction: *CrysAlis RED*; program(s) used to solve structure: *SHELXS97* (Sheldrick, 2008 ▶); program(s) used to refine structure: *SHELXL97* (Sheldrick, 2008 ▶); molecular graphics: *PLATON* (Spek, 2009 ▶); software used to prepare material for publication: *SHELXL97*.

## Supplementary Material

Crystal structure: contains datablocks I, global. DOI: 10.1107/S1600536810015394/ci5082sup1.cif

Structure factors: contains datablocks I. DOI: 10.1107/S1600536810015394/ci5082Isup2.hkl

Additional supplementary materials:  crystallographic information; 3D view; checkCIF report

## Figures and Tables

**Table 1:** Hydrogen-bond geometry (Å, °)

*D*—H⋯*A*	*D*—H	H⋯*A*	*D*⋯*A*	*D*—H⋯*A*
N1—H1*N*⋯O1^i^	0.85 (1)	2.10 (1)	2.949 (2)	174 (2)

Symmetry code: (i) 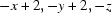.

**Fig. 1.**
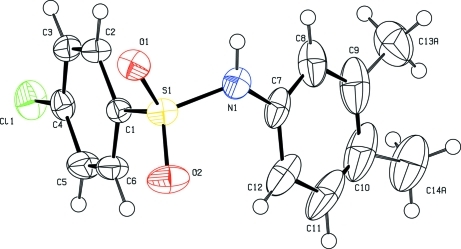
The molecular structure of (I), showing the atom labelling scheme and displacement ellipsoids are drawn at the 50% probability level. Only the major disorder component is shown.

**Fig. 2.**
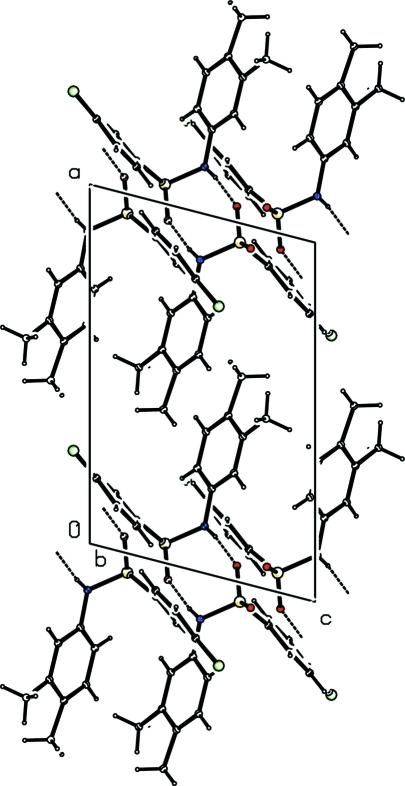
Molecular packing of (I) with hydrogen bonding shown as dashed lines.

## supplementary crystallographic information

### Comment

As part of a study of substituent effects on the structures of *N*-(aryl)arylsulfonamides (Gowda *et al.*, 2009, 2010), in the present work, the structure of 4-chloro-*N*-(3,4-dimethylphenyl)benzenesulfonamide, (I), has been determined (Fig. 1). The N1—C7 bond in the C—SO_2_—NH—C segment is gauche [C7—N1—S1—O2 = 64.1 (2)°] with respect to the S1═O1 bond and anti with respect to the S1═O2 bond [C7—N1—S1—O1 = -167.36 (18)°]. The molecule in (I) is bent at the *S*-atom with a C1—S1—N1—C7 torsion angle of -51.6 (2)°, compared to the value of -61.8 (2)° in 4-methyl-*N*-(3,4-dimethylphenyl)- benzenesulfonamide (II) (Gowda *et al.*, 2009) and 57.8 (2)° in *N*-(3,4-dimethylphenyl)benzenesulfonamide (III) (Gowda *et al.*, 2010)

The sulfonyl and the anilino benzene rings in (I) are tilted relative to each other by 65.5 (1)°, compared to the values of 47.8 (1)° in (II) and 65.4 (2)° (disordered sulfonyl ring A) and 57.8 (2)° (disordered sulfonyl ring B) in (III). The remaining bond parameters in (I) are similar to those observed in (II), (III) and other aryl sulfonamides (Perlovich *et al.*, 2006; Gelbrich *et al.*, 2007).

The crystal packing of molecules in (I) through pairs of N—H···O(S) hydrogen bonds (Table 1) is shown in Fig.2.

### Experimental

A solution of *p*-chlorobenzene (10 g) in chloroform (40 ml) was treated dropwise with chlorosulfonic acid (25 ml) at 273 K. After the initial evolution of hydrogen chloride subsided, the reaction mixture was brought to room temperature and poured into crushed ice in a beaker. The chloroform layer was separated, washed with cold water and allowed to evaporate slowly. The residual 4-chlorobenzenesulfonylchloride was treated with 3,4-dimethylaniline in the stoichiometric ratio and boiled for 10 min. The reaction mixture was then cooled to room temperature and added to ice cold water (100 ml). The resultant solid 4-chloro-*N*-(3,4-dimethylphenyl)benzenesulfonamide was filtered under suction and washed thoroughly with cold water. It was then recrystallized to constant melting point from dilute ethanol. The purity of the compound was checked and characterized by recording its infrared and NMR spectra (Shetty & Gowda, 2005). Prism like colourless single crystals used in X-ray diffraction studies were grown in ethanolic solution by slow evaporation at room temperature.

### Refinement

The H atom of the NH group was located in a difference map and refined with a distance restraint of N—H = 0.86 (1) Å. The other H atoms were positioned with idealized geometry using a riding model with C—H = 0.93–0.96 Å. All H atoms were refined with isotropic displacement parameters (set to 1.2 times of the *U*_eq_ of the parent atom).

The dimethylphenyl ring is disordered such that atom C13 moves between atoms C9 and C11. Atoms C13 and C14 were refined using a split model. The corresponding site-occupation factors were refined so that their sum was unity [0.643 (6) and 0.357 (6)]. The corresponding bond distances in the disordered groups were restrained to be equal. The U^ij^ parameters of these atoms were restrained to an approximate isotropic behavoir. Attempts to introduce disorder of the atoms C9, C10 and C11 were unsuccessful.

### Crystal data

**Table:**

C_14_H_14_ClNO_2_S	*F*(000) = 616
*M**_r_* = 295.77	*D*_x_ = 1.386 Mg m^−^^3^
Monoclinic, *P*2_1_/*c*	Mo *K*α radiation, λ = 0.71073 Å
Hall symbol: -P 2ybc	Cell parameters from 2451 reflections
*a* = 13.5000 (9) Å	θ = 2.4–27.3°
*b* = 12.4039 (8) Å	µ = 0.41 mm^−^^1^
*c* = 8.7436 (7) Å	*T* = 100 K
β = 104.492 (7)°	Prism, colourless
*V* = 1417.55 (17) Å^3^	0.44 × 0.34 × 0.22 mm
*Z* = 4	

### Data collection

**Table:**

Oxford Diffraction Xcalibur diffractometer with a Sapphire CCD detector	2890 independent reflections
Radiation source: fine-focus sealed tube	2309 reflections with *I* > 2σ(*I*)
graphite	*R*_int_ = 0.015
Rotation method data acquisition using θ and φ scans	θ_max_ = 26.4°, θ_min_ = 2.9°
Absorption correction: multi-scan (*CrysAlis RED*; Oxford Diffraction, 2009)	*h* = −16→16
*T*_min_ = 0.839, *T*_max_ = 0.915	*k* = −15→13
5943 measured reflections	*l* = −6→10

### Refinement

**Table:**

Refinement on *F*^2^	Primary atom site location: structure-invariant direct methods
Least-squares matrix: full	Secondary atom site location: difference Fourier map
*R*[*F*^2^ > 2σ(*F*^2^)] = 0.039	Hydrogen site location: inferred from neighbouring sites
*wR*(*F*^2^) = 0.107	H atoms treated by a mixture of independent and constrained refinement
*S* = 1.07	*w* = 1/[σ^2^(*F*_o_^2^) + (0.0536*P*)^2^ + 0.6002*P*] where *P* = (*F*_o_^2^ + 2*F*_c_^2^)/3
2890 reflections	(Δ/σ)_max_ = 0.045
194 parameters	Δρ_max_ = 0.31 e Å^−^^3^
28 restraints	Δρ_min_ = −0.24 e Å^−^^3^

### Special details

**Table:**

Geometry. All esds (except the esd in the dihedral angle between two l.s. planes) are estimated using the full covariance matrix. The cell esds are taken into account individually in the estimation of esds in distances, angles and torsion angles; correlations between esds in cell parameters are only used when they are defined by crystal symmetry. An approximate (isotropic) treatment of cell esds is used for estimating esds involving l.s. planes.
Refinement. Refinement of *F*^2^ against ALL reflections. The weighted *R*-factor wR and goodness of fit *S* are based on *F*^2^, conventional *R*-factors *R* are based on *F*, with *F* set to zero for negative *F*^2^. The threshold expression of *F*^2^ > σ(*F*^2^) is used only for calculating *R*-factors(gt) etc. and is not relevant to the choice of reflections for refinement. *R*-factors based on *F*^2^ are statistically about twice as large as those based on *F*, and *R*- factors based on ALL data will be even larger.

### Fractional atomic coordinates and isotropic or equivalent isotropic displacement parameters (Å^2^)

**Table:**

	*x*	*y*	*z*	*U*_iso_*/*U*_eq_	Occ. (<1)
C1	0.90057 (14)	0.96543 (15)	0.2942 (2)	0.0261 (4)	
C2	0.91759 (15)	1.07570 (16)	0.2922 (2)	0.0321 (4)	
H2	0.9592	1.1047	0.2291	0.039*	
C3	0.87384 (16)	1.14279 (16)	0.3821 (2)	0.0327 (5)	
H3	0.8856	1.2183	0.3832	0.039*	
C4	0.81360 (15)	1.09927 (17)	0.4694 (2)	0.0318 (4)	
C5	0.79804 (19)	0.98936 (18)	0.4760 (3)	0.0414 (5)	
H5	0.7575	0.9609	0.5409	0.050*	
C6	0.84233 (17)	0.92178 (17)	0.3870 (2)	0.0361 (5)	
H6	0.8327	0.8460	0.3897	0.043*	
C7	0.75973 (16)	0.8915 (2)	−0.0415 (3)	0.0441 (6)	
C8	0.69977 (19)	0.9665 (3)	−0.1406 (3)	0.0586 (7)	
H8	0.7317	1.0233	−0.1833	0.070*	
C9	0.5922 (2)	0.9596 (3)	−0.1791 (4)	0.0778 (10)	
H9	0.5524	1.0104	−0.2499	0.093*	0.357 (6)
C10	0.5445 (2)	0.8797 (4)	−0.1148 (5)	0.0887 (13)	
C11	0.6051 (3)	0.8072 (3)	−0.0163 (5)	0.0808 (12)	
H11	0.5728	0.7518	0.0287	0.097*	0.643 (6)
C12	0.7124 (2)	0.8098 (2)	0.0221 (3)	0.0588 (8)	
H12	0.7516	0.7570	0.0899	0.071*	
C13A	0.5254 (3)	1.0313 (5)	−0.2823 (6)	0.0755 (17)	0.643 (6)
H13A	0.4543	1.0100	−0.2906	0.091*	0.643 (6)
H13B	0.5392	1.0286	−0.3870	0.091*	0.643 (6)
H13C	0.5364	1.1048	−0.2405	0.091*	0.643 (6)
C14A	0.4264 (5)	0.8913 (8)	−0.1730 (11)	0.098 (3)	0.643 (6)
H14A	0.4056	0.8831	−0.2881	0.117*	0.643 (6)
H14B	0.4057	0.9626	−0.1439	0.117*	0.643 (6)
H14C	0.3933	0.8354	−0.1238	0.117*	0.643 (6)
N1	0.86904 (13)	0.89945 (15)	−0.0097 (2)	0.0360 (4)	
H1N	0.8902 (18)	0.9510 (14)	−0.057 (3)	0.043*	
O1	1.04407 (10)	0.92002 (12)	0.15306 (17)	0.0381 (4)	
O2	0.93722 (13)	0.77276 (12)	0.2139 (2)	0.0474 (4)	
Cl1	0.75301 (5)	1.18581 (5)	0.57252 (7)	0.04891 (19)	
S1	0.94625 (4)	0.88149 (4)	0.16631 (6)	0.03178 (16)	
C13B	0.5471 (7)	0.7346 (8)	0.0226 (12)	0.081 (3)	0.357 (6)
H13D	0.5895	0.6822	0.0938	0.097*	0.357 (6)
H13E	0.5083	0.6976	−0.0727	0.097*	0.357 (6)
H13F	0.4996	0.7690	0.0759	0.097*	0.357 (6)
C14B	0.4335 (8)	0.8452 (11)	−0.1288 (16)	0.068 (3)	0.357 (6)
H14D	0.3982	0.8363	−0.2405	0.082*	0.357 (6)
H14E	0.3986	0.9005	−0.0816	0.082*	0.357 (6)
H14F	0.4328	0.7767	−0.0733	0.082*	0.357 (6)

### Atomic displacement parameters (Å^2^)

**Table:**

	*U*^11^	*U*^22^	*U*^33^	*U*^12^	*U*^13^	*U*^23^
C1	0.0273 (9)	0.0242 (9)	0.0267 (9)	0.0021 (7)	0.0063 (7)	0.0004 (8)
C2	0.0347 (10)	0.0279 (10)	0.0360 (11)	−0.0018 (8)	0.0132 (9)	0.0033 (8)
C3	0.0381 (11)	0.0227 (10)	0.0354 (11)	0.0013 (8)	0.0057 (9)	0.0001 (8)
C4	0.0360 (11)	0.0337 (11)	0.0246 (9)	0.0057 (8)	0.0058 (8)	−0.0054 (8)
C5	0.0579 (14)	0.0374 (12)	0.0368 (12)	−0.0042 (10)	0.0265 (11)	−0.0021 (10)
C6	0.0519 (13)	0.0252 (10)	0.0359 (11)	−0.0019 (9)	0.0201 (10)	0.0013 (9)
C7	0.0324 (11)	0.0642 (16)	0.0390 (12)	−0.0098 (10)	0.0153 (9)	−0.0290 (12)
C8	0.0390 (13)	0.096 (2)	0.0401 (13)	0.0017 (13)	0.0079 (11)	−0.0184 (14)
C9	0.0402 (15)	0.130 (3)	0.0553 (17)	0.0094 (18)	−0.0033 (13)	−0.028 (2)
C10	0.0359 (15)	0.141 (4)	0.089 (2)	−0.026 (2)	0.0145 (17)	−0.056 (3)
C11	0.0532 (18)	0.102 (3)	0.099 (3)	−0.0467 (19)	0.0412 (18)	−0.059 (2)
C12	0.0484 (14)	0.0672 (18)	0.0686 (18)	−0.0250 (13)	0.0292 (13)	−0.0355 (15)
C13A	0.043 (2)	0.114 (4)	0.065 (3)	0.005 (2)	0.005 (2)	0.016 (3)
C14A	0.043 (3)	0.123 (6)	0.114 (6)	−0.017 (4)	−0.003 (3)	−0.029 (5)
N1	0.0312 (9)	0.0456 (11)	0.0351 (10)	−0.0043 (8)	0.0158 (7)	−0.0083 (8)
O1	0.0279 (7)	0.0451 (9)	0.0442 (9)	0.0049 (6)	0.0146 (6)	0.0041 (7)
O2	0.0592 (10)	0.0260 (8)	0.0676 (11)	0.0074 (7)	0.0355 (9)	0.0023 (8)
Cl1	0.0579 (4)	0.0490 (4)	0.0418 (3)	0.0103 (3)	0.0163 (3)	−0.0153 (3)
S1	0.0315 (3)	0.0293 (3)	0.0384 (3)	0.0036 (2)	0.0160 (2)	−0.0004 (2)
C13B	0.065 (5)	0.084 (6)	0.097 (6)	−0.031 (4)	0.026 (4)	0.016 (5)
C14B	0.036 (5)	0.094 (7)	0.075 (6)	−0.032 (5)	0.016 (4)	0.012 (5)

### Geometric parameters (Å, °)

**Table:**

C1—C6	1.374 (3)	C10—C14A	1.554 (7)
C1—C2	1.388 (3)	C11—C13B	1.293 (8)
C1—S1	1.7490 (19)	C11—C12	1.403 (4)
C2—C3	1.376 (3)	C11—H11	0.95
C2—H2	0.95	C12—H12	0.95
C3—C4	1.358 (3)	C13A—H13A	0.98
C3—H3	0.95	C13A—H13B	0.98
C4—C5	1.383 (3)	C13A—H13C	0.98
C4—Cl1	1.733 (2)	C14A—H14A	0.98
C5—C6	1.378 (3)	C14A—H14B	0.98
C5—H5	0.95	C14A—H14C	0.98
C6—H6	0.95	N1—S1	1.6439 (19)
C7—C8	1.385 (4)	N1—H1N	0.849 (10)
C7—C12	1.387 (4)	O1—S1	1.4360 (14)
C7—N1	1.435 (3)	O2—S1	1.4255 (16)
C8—C9	1.409 (4)	C13B—H13D	0.98
C8—H8	0.95	C13B—H13E	0.98
C9—C10	1.377 (6)	C13B—H13F	0.98
C9—C13A	1.418 (5)	C14B—H14D	0.98
C9—H9	0.95	C14B—H14E	0.98
C10—C11	1.367 (6)	C14B—H14F	0.98
C10—C14B	1.533 (10)		
			
C6—C1—C2	121.11 (18)	C12—C11—H11	118.1
C6—C1—S1	119.21 (15)	C7—C12—C11	118.2 (3)
C2—C1—S1	119.53 (15)	C7—C12—H12	120.9
C3—C2—C1	119.54 (19)	C11—C12—H12	120.9
C3—C2—H2	120.2	C9—C13A—H13A	109.5
C1—C2—H2	120.2	C9—C13A—H13B	109.5
C4—C3—C2	118.92 (19)	H13A—C13A—H13B	109.5
C4—C3—H3	120.5	C9—C13A—H13C	109.5
C2—C3—H3	120.5	H13A—C13A—H13C	109.5
C3—C4—C5	122.28 (19)	H13B—C13A—H13C	109.5
C3—C4—Cl1	118.22 (16)	C10—C14A—H14A	109.5
C5—C4—Cl1	119.49 (17)	C10—C14A—H14B	109.5
C6—C5—C4	118.9 (2)	H14A—C14A—H14B	109.5
C6—C5—H5	120.5	C10—C14A—H14C	109.5
C4—C5—H5	120.5	H14A—C14A—H14C	109.5
C1—C6—C5	119.14 (19)	H14B—C14A—H14C	109.5
C1—C6—H6	120.4	C7—N1—S1	123.52 (15)
C5—C6—H6	120.4	C7—N1—H1N	114.3 (17)
C8—C7—C12	119.1 (2)	S1—N1—H1N	110.2 (17)
C8—C7—N1	119.1 (2)	O2—S1—O1	119.07 (10)
C12—C7—N1	121.8 (2)	O2—S1—N1	108.71 (11)
C7—C8—C9	121.0 (3)	O1—S1—N1	105.06 (9)
C7—C8—H8	119.5	O2—S1—C1	107.89 (9)
C9—C8—H8	119.5	O1—S1—C1	109.38 (9)
C10—C9—C8	120.4 (3)	N1—S1—C1	105.98 (9)
C10—C9—C13A	115.0 (3)	C11—C13B—H13D	109.5
C8—C9—C13A	124.6 (4)	C11—C13B—H13E	109.5
C10—C9—H9	119.8	H13D—C13B—H13E	109.5
C8—C9—H9	119.8	C11—C13B—H13F	109.5
C11—C10—C9	117.6 (3)	H13D—C13B—H13F	109.5
C11—C10—C14B	106.6 (6)	H13E—C13B—H13F	109.5
C9—C10—C14B	135.8 (6)	C10—C14B—H14D	109.5
C11—C10—C14A	132.1 (5)	C10—C14B—H14E	109.5
C9—C10—C14A	110.3 (5)	H14D—C14B—H14E	109.5
C13B—C11—C10	108.5 (6)	C10—C14B—H14F	109.5
C13B—C11—C12	127.6 (6)	H14D—C14B—H14F	109.5
C10—C11—C12	123.7 (3)	H14E—C14B—H14F	109.5
C10—C11—H11	118.1		
			
C6—C1—C2—C3	−1.3 (3)	C14B—C10—C11—C13B	2.7 (9)
S1—C1—C2—C3	174.13 (15)	C14A—C10—C11—C13B	4.3 (8)
C1—C2—C3—C4	−0.9 (3)	C9—C10—C11—C12	−0.1 (5)
C2—C3—C4—C5	2.7 (3)	C14B—C10—C11—C12	178.0 (7)
C2—C3—C4—Cl1	−176.19 (15)	C14A—C10—C11—C12	179.6 (5)
C3—C4—C5—C6	−2.2 (3)	C8—C7—C12—C11	−0.5 (3)
Cl1—C4—C5—C6	176.64 (17)	N1—C7—C12—C11	−179.3 (2)
C2—C1—C6—C5	1.8 (3)	C13B—C11—C12—C7	175.4 (7)
S1—C1—C6—C5	−173.69 (17)	C10—C11—C12—C7	1.0 (4)
C4—C5—C6—C1	0.0 (3)	C8—C7—N1—S1	138.1 (2)
C12—C7—C8—C9	−1.0 (4)	C12—C7—N1—S1	−43.0 (3)
N1—C7—C8—C9	177.9 (2)	C7—N1—S1—O2	64.1 (2)
C7—C8—C9—C10	1.9 (4)	C7—N1—S1—O1	−167.36 (18)
C7—C8—C9—C13A	−178.5 (4)	C7—N1—S1—C1	−51.6 (2)
C8—C9—C10—C11	−1.4 (5)	C6—C1—S1—O2	−16.05 (19)
C13A—C9—C10—C11	179.0 (4)	C2—C1—S1—O2	168.43 (16)
C8—C9—C10—C14B	−178.7 (9)	C6—C1—S1—O1	−146.96 (16)
C13A—C9—C10—C14B	1.7 (10)	C2—C1—S1—O1	37.52 (18)
C8—C9—C10—C14A	178.8 (4)	C6—C1—S1—N1	100.26 (17)
C13A—C9—C10—C14A	−0.8 (6)	C2—C1—S1—N1	−75.27 (17)
C9—C10—C11—C13B	−175.4 (6)		

### Hydrogen-bond geometry (Å, °)

**Table:**

*D*—H···*A*	*D*—H	H···*A*	*D*···*A*	*D*—H···*A*
N1—H1N···O1^i^	0.85 (1)	2.10 (1)	2.949 (2)	174 (2)

Symmetry codes: (i) −*x*+2, −*y*+2, −*z*.
